# Miliary Tuberculosis Presenting with Hyponatremia and ARDS in an 82-Year-Old Immunocompetent Female

**DOI:** 10.3390/pathogens7030072

**Published:** 2018-09-05

**Authors:** Benjamín Herreros, Isabel Plaza, Rebeca García, Marta Chichón, Carmen Guerrero, Emilio Pintor

**Affiliations:** 1 Department of Internal Medicine, Hospital Universitario Fundación Alcorcón, 28922 Madrid, Spain; BHerreros@fhalcorcon.es (B.H.); rebecagarciacaballero@gmail.com (R.G.); mchichon@fhalcorcon.es (M.C.); 2 School of Biomedical and Health Sciences, Universidad Europea de Madrid, 28670 Madrid, Spain; 3 Department of Nuclear Medicine, Hospital Puerta de Hierro, 28222 Madrid, Spain; iplazaheras@hotmail.com; 4 Department of Pathology, Hospital Universitario Fundación Alcorcón, 28922 Madrid, Spain; cguerrero@fhalcorcon.es

**Keywords:** miliary tuberculosis, acute respiratory distress syndrome (ARDS), hyponatremia, necropsy

## Abstract

An immunocompetent 82-year-old female was admitted to our hospital due to fever without clear origin and hyponatremia. In the following days, an acute and bilateral pulmonary infiltrate appeared with a progressive worsening in respiratory function. Chest x-ray and CT (Computed tomography) showed bilateral reticulonodular infiltrates. Bronchoscopic aspiration and bronchoalveolar lavage (BAL), and transbronchial lung biopsy (TBBX) studies did not reveal microbiological and histopathological diagnosis. Broad-spectrum antibiotics were non-effective, and the patient died due to respiratory failure. Necropsy study revealed a miliary tuberculosis affecting lungs, liver, bone marrow, spleen, kidney, arteries, pancreas, and adrenal glands. Some weeks after the patient´s death, mycobacterial cultures from sputum, BAL and TBBX samples were positive for *Mycobacterium tuberculosis*.

## 1. Introduction

Miliary tuberculosis (TB) results from a massive lymphohematogenous dissemination of *Mycobacterium tuberculosis* bacilli and is characterized by tiny tubercles evident on gross pathology resembling millet seeds in size and appearance [1]. Considered to be predominantly a disease of infants and children in the pre-antibiotic era, miliary TB is increasingly being encountered in adults as well. Among reasons that justify these epidemiological changes in miliary tuberculosis, we can find an increased number of immunocompromised patients due to the HIV-AIDS pandemic and also due to therapy with different types of immunosuppressive drugs. Although uncommon, sometimes it can happen in immunocompetent patients.

Clinical manifestations of miliary TB are protean and non-specific, such as anorexia, prolonged fever, and weight loss. When lungs are affected, it is common to present dyspnea and productive cough. Atypical clinical presentation often delays the diagnosis. Although uncommon, miliary TB can cause acute respiratory distress syndrome (ARDS) [2,3] in patients with extensive pulmonary parenchymal involvement. Disseminated pulmonary micronodules (miliary pattern) is typical radiological presentation, but in some patients, other radiological patterns can appear. Clinicians, therefore, should have a low threshold for suspecting miliary TB.

We report a rare presentation of miliary tuberculosis as ARDS with hyponatremia in an old immunocompetent female.

## 2. Case Report

An 82-year-old female with a previous medical history (PMH) of type 2 diabetes mellitus, chronic atrial fibrillation, and several cardio-embolic lacunar strokes on chronic therapy with apixaban was admitted to the emergency department with a six-day history of a fever, chills, and general malaise. Her general practitioner prescribed her empiric therapy with amoxicillin-clavulanic with no improvement. She was vaccinated against *Influenza* and *Pneumoccocus* every year, but she had never received a BCG (Bacillus Calmette-Guerin) vaccine. She lived alone, and nearest family members were asymptomatic.

**Figure 1:**
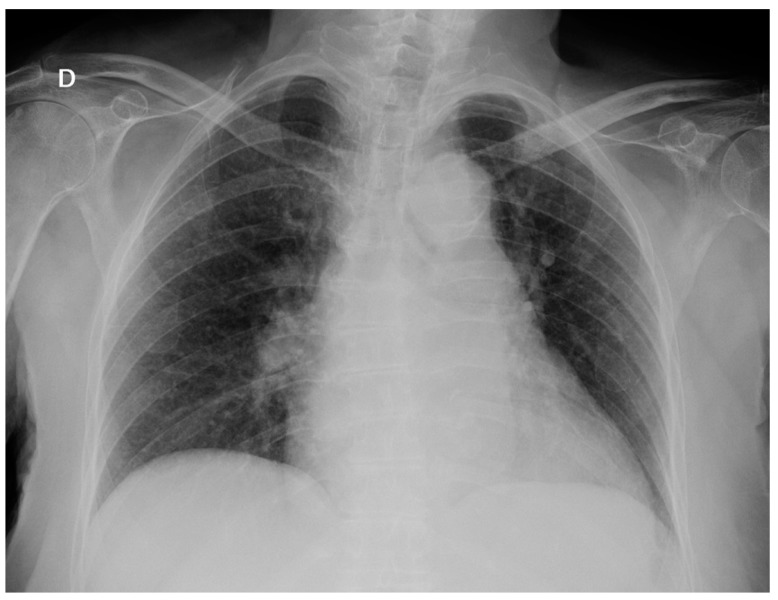
Chest X-ray on admission: there are no infiltrates or opacities.

At admission, she was febrile (39 °C), eupneic with oxygen saturation 99%, a blood pressure of 125/85 mmHg, with a pulse rate of 86 bpm, arrhythmic, and a breath rate of 14 bpm. Physical exams revealed no abnormalities. At emergency department evaluation, laboratory work-up results were as follows: 6300 leukocytes with 80% neutrophils, erythrocytes count, platelets count, electrolytes and biochemistry tests were normal except for glycemia: 155 mg/dl, natremia: 123 nmol/L and C-reactive protein: 87 mg/dl. Urine analysis: 100 leukocytes and 10 erythrocytes per high-power field. Chest X-ray: normal without pulmonary infiltrates (Figure 1).

A urinary tract infection (UTI) was suspected in this patient besides hyponatremia, and ciprofloxacin was prescribed. On the fourth day, the patient continued with fever and meropemen was administered instead of ciprofloxacin. All microbiological studies at that moment were negative including blood and urine cultures, Mantoux test, PCR for influenza virus and respiratory syncytial virus and TSH: 1.8 µU/mL and plasmatic cortisol: 25.5 µg/dl. Echocardiogram and abdominal ultrasound were also normal.

**Figure 2:**
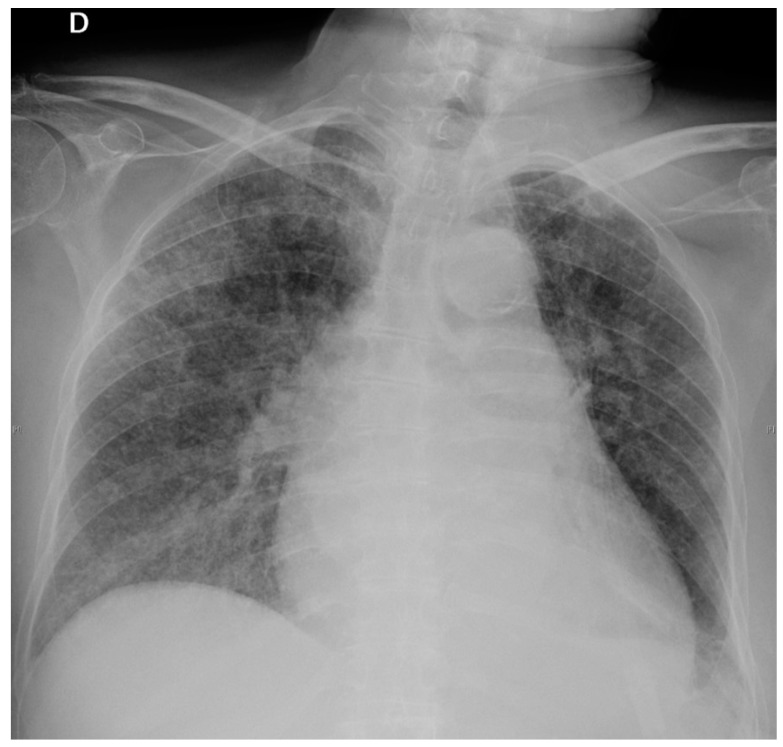
Chest X-ray on 7th day: Bilateral interstitial infiltrates.

Three days later, the patient started with progressive dyspnea with productive cough. Arterial blood gas analysis showed PO2: 67 mmHg, PCO2: 39 mmHg and SatO2: 94% and a new chest X-ray (Figure 2) presented bilateral interstitial infiltrate. Sputum culture and acid-fast stain were negative. Urinary *Legionella* antigen was also negative.

**Figure 3:**
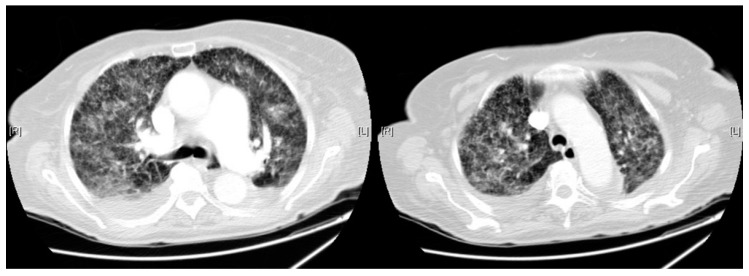
Chest CT on 10th day: Extensive pulmonary parenchymal involvement consisting of irregular septal thickenings with ground-glass areas and centrilobular nodules with a peri-lymphatic distribution.

Chest CT scan revealed an extensive pulmonary parenchymal involvement consisting of irregular septal thickenings with ground-glass areas and centrilobular nodules with a peri-lymphatic distribution, which had a predominantly central distribution although it affected the entire parenchyma of both hemithorax without apico-basal gradient (Figure 3).

A fiberoptic bronchoscopy with bronchoalveolar lavage (BAL) and transbronchial lung biopsy (TBBX) were performed. Cytology from BAL revealed inflammatory cells. Microbiological studies: negative culture, Ziehl-Neelsen stain negative, IFA (immunofluorescence assay) for *P. jirovecii* negative. TBBX showed no positive histopathological or microbiological results. Serology was negative for HIV (Human Immunodeficiency virus), Cytomegalovirus (CMV), Herpes Virus 6 (HV 6), Epstein Barr virus (EBV) and *Coxiella burneti*.

**Figure 4:**
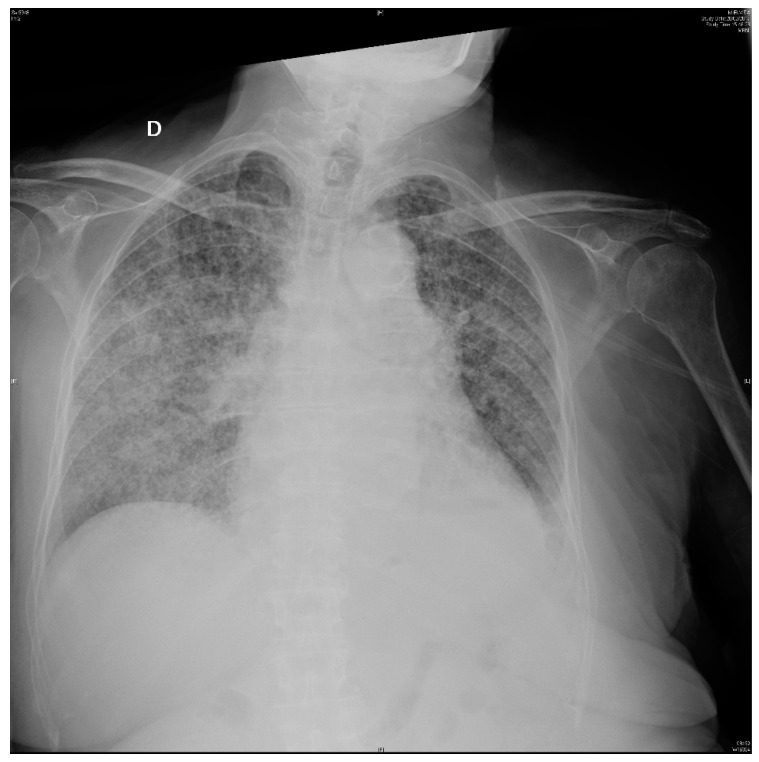
Chest X-ray on 16th day: Extensive bilateral reticulo-nodular infiltrates.

After bronchoscopy, steroids were administered (methylprednisolone 0.5 mg/kg) but respiratory distress increased (Figure 4) and the patient died five days later.

Shortly after her demise, results from her sputum mycobacterial culture in Lowenstein-Nielsen medium; on day seventh, they tested positive for *Mycobacterium tuberculosis*. Some weeks later, results from BAL and TBBX mycobacterial cultures were also positive in both samples.

**Figure 5:**
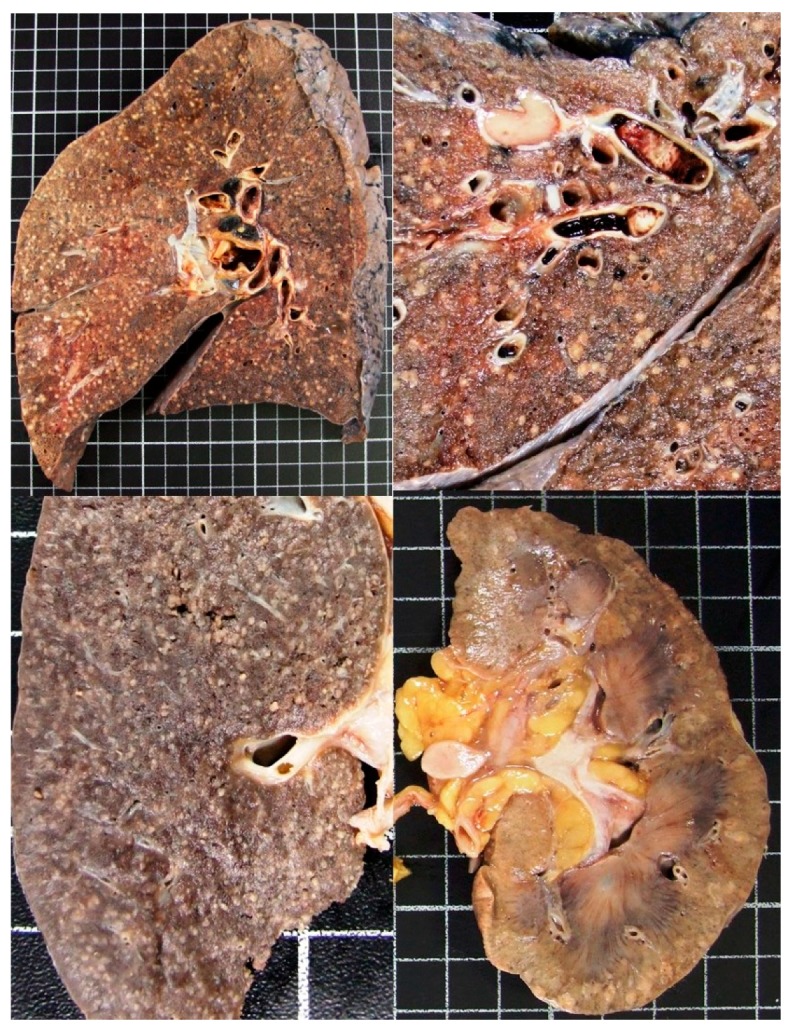
Macroscopic appearance at necropsy: Cut section of the lungs (upper images), spleen and kidney (lower images) shows micronodules (1–4 mm in diameter) which resemble millet seeds.

The complete necropsy was performed to detect cause of death and pulmonary pathology. Macroscopic appearance showed multiple white-yellowish lesions 1 to 4 mm in diameter that resemble millet or rice seed, affecting lungs, liver, spleen, kidney, lymph nodes, pancreas, and adrenal glands (Figure 5).

**Figure 6:**
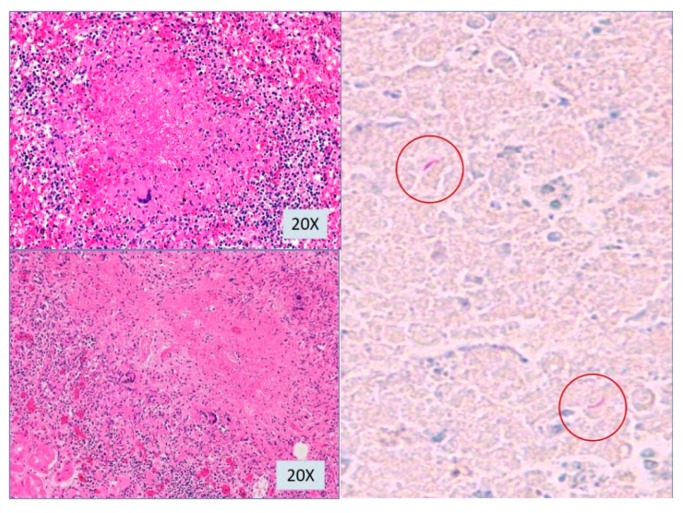
Microscopic examination: epithelioid granuloma with multinucleated giant cells (H & E) from kidney and spleen micronodules (left images) with acid-fast bacilli (ZN (Ziehl-Neelsen) stain) (right image).

Microscopic studies revealed all those white-yellowish nodules contained central caseating granulomas with several acid-fast bacilli on Ziehl-Neelsen stanning (Figure 6). There were also granulomas inside bone marrow and in some arterial walls. Miliary lesions were not found in the necropsic study of the central nervous system.

## 3. Discussion

Miliary TB results from a massive lympho-hematogenous dissemination of *Mycobacterium tuberculosis*. Although it has been considered to be a childhood disease, over the past three decades, it is increasingly being recognized in adults as well. Reasons for this changing epidemiological trend include: the HIV epidemic, and ever-increasing list of causes of immunosuppression (therapy with steroids, immunosuppressive and cytotoxic drugs) [1]. In recent decades, use of immunomodulatory drugs such as anti-tumor necrosis factors (TNF) agents (infliximab, etanercept and adalimulab) also increases risk for developing miliary TB [4]. Among immunocompetent adults, miliary TB accounts for less than 2% of all cases of TB and up to 20% of all extra pulmonary TB [4].

The clinical manifestation of miliary TB in adults are protean and non-specific, and can be obscure till late in the disease. It makes diagnosing a challenge that can perplex even the most experienced clinicians and mortality has remained high despite effective therapy being available. Most common symptomatology at initial presentation in adults is non-specific such as anorexia, fever, weight loss, night sweat and chills or respiratory symptoms such as dyspnea, chest pain and productive cough [4,5] Upon physical examination, it is common to find respiratory signs and hepatomegaly. Less frequent signs are splenomegaly, neurological signs, ascites, and choroidal tubercles upon funduscopic examination. Sometimes, miliary TB can present as ARDS. It represents less than 8% of cases and it is associated with very high mortality (40–80%) despite mechanical ventilation and corticosteroids [6,7,8,9,10].

Tuberculin anergy is more common in miliary TB than in pulmonary and extra pulmonary TB [11]; Mantoux test conversion may occur following successful therapy. T-cell-based interferon-gamma release assay (IGRA) is a recent test to study in patients with miliary TB. Although a positive IGRA test does not distinguish between active or latent TB, a negative IGRA tests result can be useful for ruling out a diagnosis of TB. Several hematological and biochemical abnormalities are known to happen in miliary TB. The most common ones are anemia and elevated ESR (Erythrocyte Sedimentation Rate) and CRP (C-Reactive Protein) and changes in some plasmatic electrolyte levels: One of them is hyponatremia. It occurs in up to 50% of patients with pulmonary tuberculosis. It can occur due to dysregulation in ADH (Antidiuretic hormone) release or due to Addison´s disease [11,12,13]. In our patient, the most likely reason is adrenal involvement.

Chest radiography can be useful in the diagnosis of miliary TB. Radiological classical presentation (50%) consists of the presence of small nodules (1–4 mm in diameter) scattered through both lungs (miliary pattern). However, x-rays may appear to be normal in the early stage of the disease or with other radiologic patterns: interstitial, reticulonodular or even as pleural effusion; therefore, a diagnosis of miliary TB from chest x-ray can be difficult. Several studies have shown that CT imaging is more sensitive for miliary tuberculosis diagnosis. Some researchers have described CT features [14]: miliary nodules, ground-glass attenuation, and reticular opacity, having some imaging differences between patients with or without HIV [15]. Other imaging studies have been tested in patients with miliary TB, such as pulmonary ultrasound [16] or MRI, with diagnostic results not superior to CT. Our patient presented a radiological pattern: reticulonodular bilateral infiltrate (not typical miliary pattern), which made us consider a differential diagnosis including other microorganisms responsible for producing this type of radiological appearance such as bacterial (Mycoplasma and Legionella), viral (Respiratory syncytial virus, influenza virus and CMV), or fungal (*Aspergillus*, *candida*, and *P. jirovecii*).

In patients with suspected miliary TB, depending on the organ involved, appropriate samples must be obtained to confirm histopathological and/or microbiological diagnosis [1]. Diagnosis can be achieved before a patient´s death with several fluid cultures or tissues. Most common positive fluids are sputum (41.4%), bronchoscopy aspirate (46.8%), CSF (21.2%) or urine (32.7%). Among tissue biopsy, diagnosis can be positive with bone marrow (66.7%); liver (88.9%) or lymph node (90.9%). TBBX samples are a good way to achieve diagnosis in miliary tuberculosis when chest X-ray shows a typical miliary pattern. In this situation, granulomatous lesions suggestive of TB can be found in up to 60% of cases [17]. Unfortunately, an important number of patients with miliary TB will not be diagnosed before a patient’s death, and it will be confirmed during autopsy [18,19,20,21].

Regarding treatment, patients with miliary TB must be promptly treated with standard anti-tuberculosis therapy, as this disease will be lethal if not treated [22]. However, there is no consensus about optimum duration of treatment. Adjunctive corticosteroid treatment in patients with miliary TB is considered to be beneficial in some circumstances such as TB meningitis, ARDS [8], large pericardial effusion or Addison’s disease [23].

Mortality related to miliary TB is high, reaching 25–30% of all adult cases. Delay in diagnosis, or no diagnosis, and consequently, delayed starting in specific anti-TB therapy appear to be the most important factor responsible for this high mortality rate.

Although severe complications of miliary tuberculosis are frequent, mortality can be lower where access to critical care intervention, anti-tuberculous therapy and corticosteroid use are possible [8,9,10].

This case highlights an atypical presentation of miliary tuberculosis in an old and immunocompetent female in a western and developed country with hyponatremia, a bilateral pulmonary interstitial infiltrate that produced her ARDS and death.
